# Supplementary figures and images for: Function and contribution of two putative Enterococcus faecalis glycosaminoglycan degrading enzymes to bacteremia and catheter-associated urinary tract infection
Source: Infect Immun. 2024 Jun 6;92(7):e00199-24. doi: 10.1128/iai.00199-24 (PMC11238560; doi:10.1128/iai.00199-24)

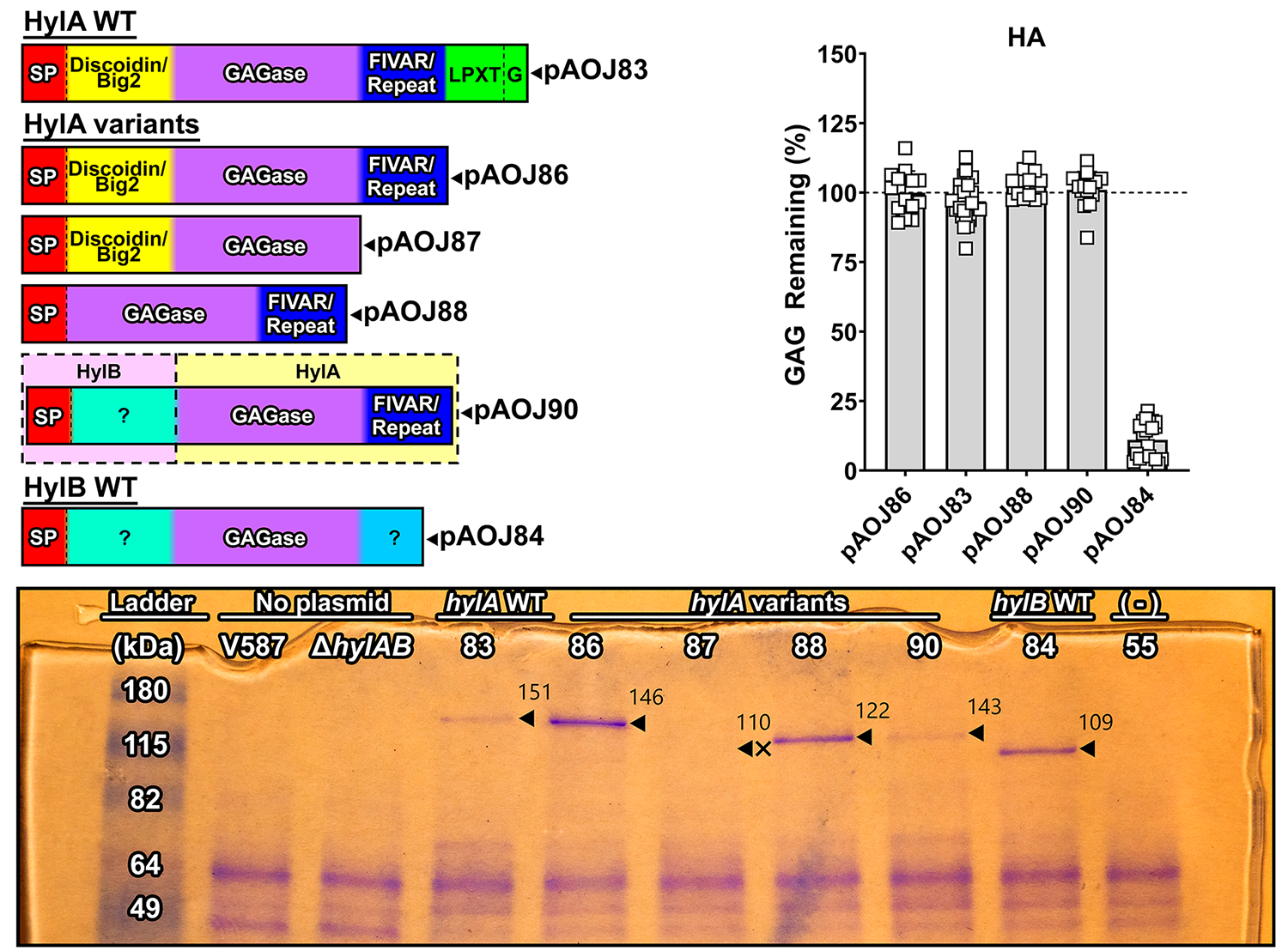

Supplement: Figure S1 — HylA and HylB constructs utilized in this study and their secretion profiles. [file iai.00199-24-s0001.tif]

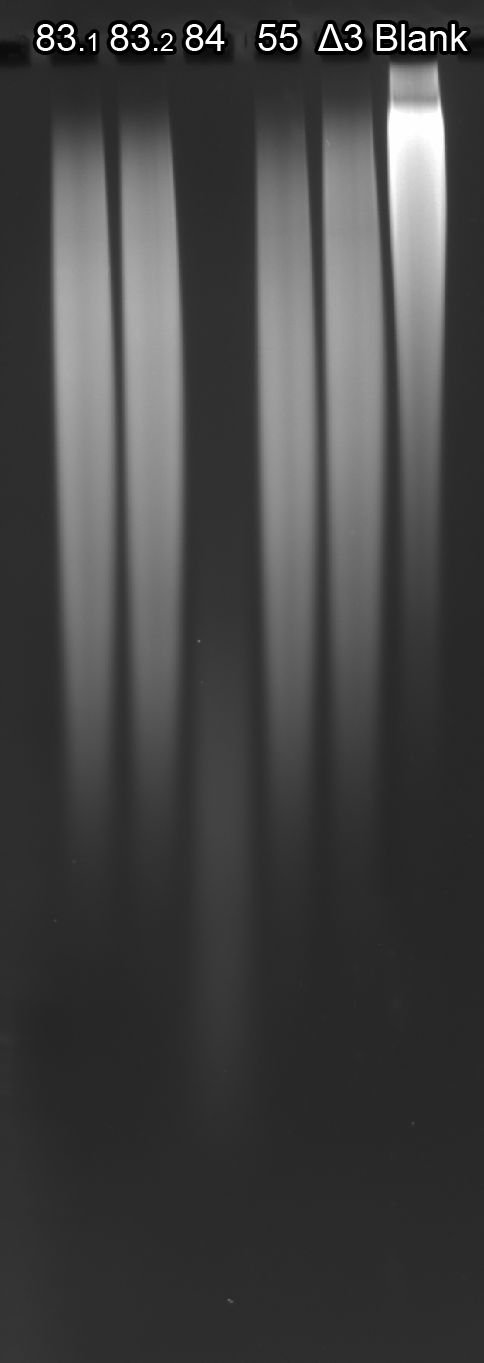

Supplement: Figure S2 — Deleting ef2268 does not impact basal HA digestion. [file iai.00199-24-s0002.tif]

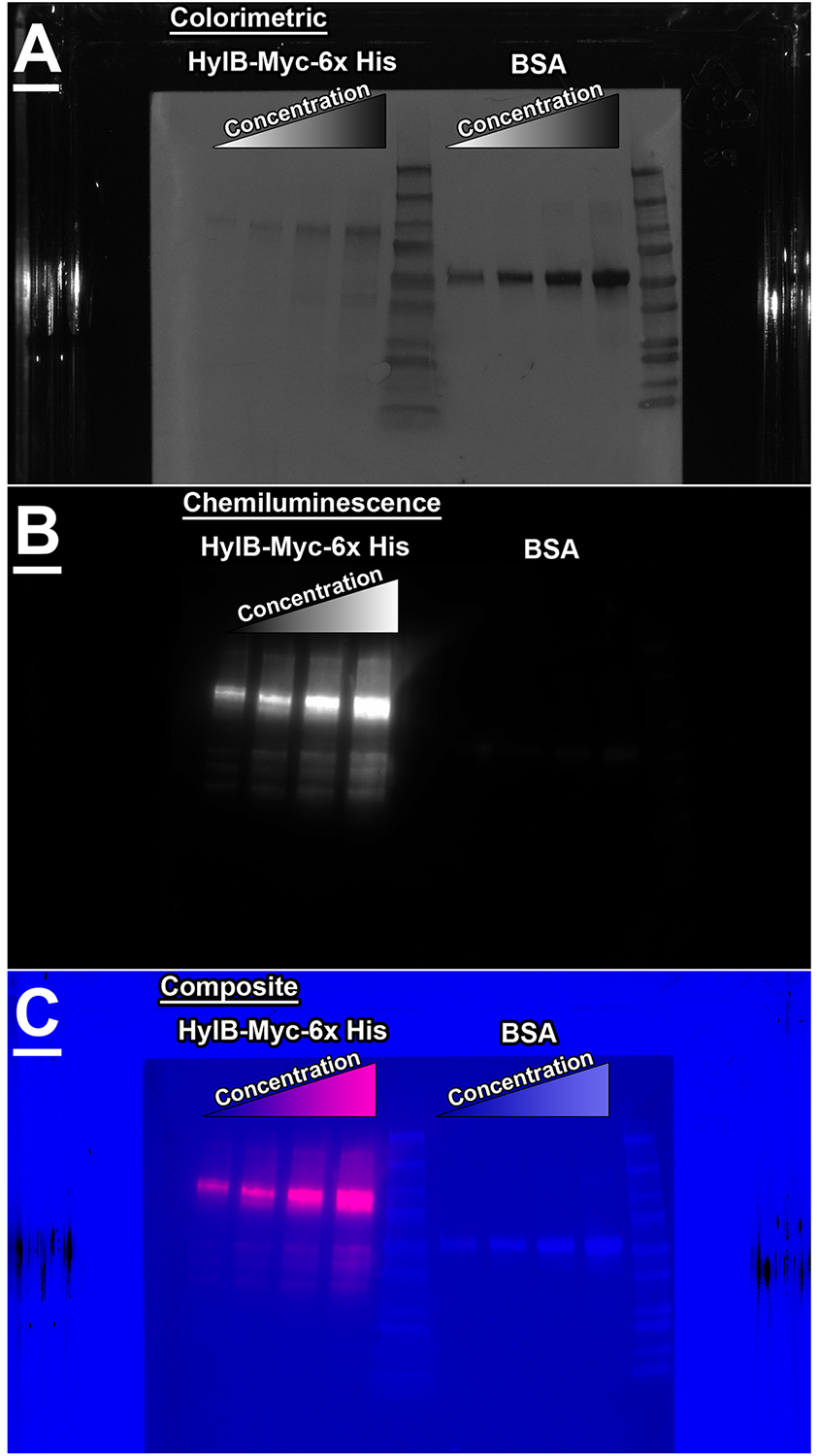

Supplement: Figure S3 — Determination of recombinant HylB concentration and purity. [file iai.00199-24-s0003.tif]

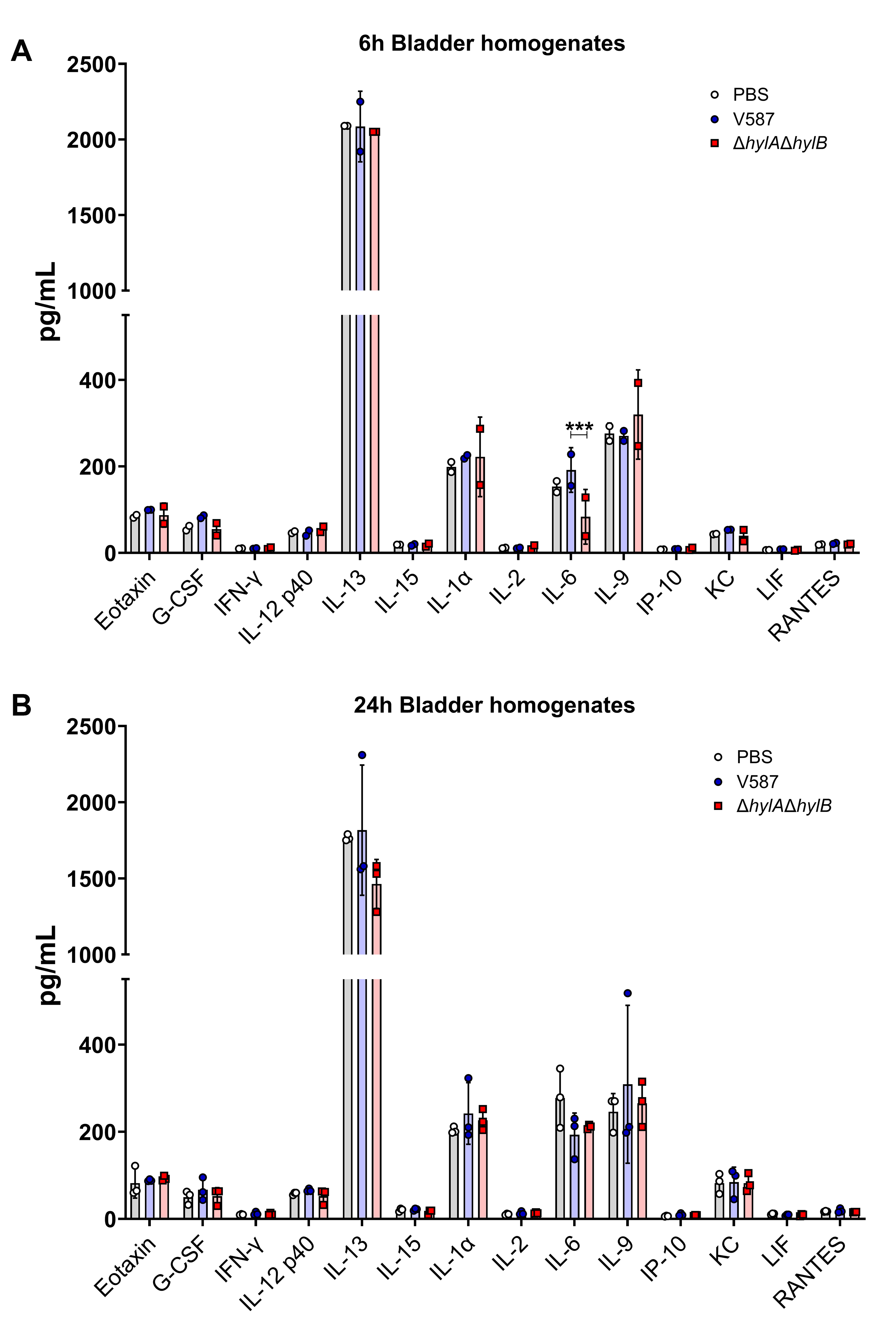

Supplement: Figure S4 — Bladder cytokine and chemokine profiles during E. faecalis CAUTI. [file iai.00199-24-s0004.tif]
