# Supplementary material for: Function and contribution of two putative Enterococcus faecalis glycosaminoglycan degrading enzymes to bacteremia and catheter-associated urinary tract infection
Source: Infect Immun. 2024 Jun 6;92(7):e00199-24. doi: 10.1128/iai.00199-24 (PMC11238560; doi:10.1128/iai.00199-24)
Supplement: Supplemental legends — Legends for Figures S1 to S4. [file iai.00199-24-s0005.docx]

**Supplemental Figure 1. HylA and HylB constructs utilized in this study and their secretion profiles.** (A) All HylA and HylB constructs cloned into pAOJ20 for expression in *E. faecalis* Δ*hylA*Δ*hylB*. SP indicates a signal peptide as predicted by SignalP 5.0. A dashed line indicates a predicted proteolytic cleavage site with either signal peptidase (SP) or sortase (LPXTG). The FIVAR/Repeat domain indicates a complete 69 amino acid segment with a predicted “FIVAR” domain, followed by three 71 amino acid repeats with partial FIVAR domain prediction. “?” indicates a stretch of >40 amino acids which have no predicted conserved domains or other obvious features. pAOJ90 contains the N-terminal region of HylB (pink) fused to the C terminal region of HylA (yellow), as shown by the dashed boxes. (B) HA degradation by Δ*hylA*Δ*hylB* transformed with the indicated plasmids in the semi-quantitative assay. Error bars represent mean and standard deviation for at least three independent experiments with at least two replicates each. (C) Coomassie Brilliant Blue stained SDS-PAGE gels of soluble secreted protein from cell-free supernatants . Lanes are labeled with strain names, with numbers to indicate the plasmid name of the HylA and HylB constructs expressed in Δ*hylA*Δ*hylB*. Numbers accompanied by arrows are the predicted molecular weight, in kilodaltons (kDa), of a given plasmid-encoded protein. Both gels are representative of at least two independent experiments per strain. Photographs were taken using a Nikon Z30, then adjusted using Adobe Photoshop 2022 for exposure, levels, hue, saturation and/or contrast until fainter bands, easily visible by eye, were also distinguishable from background in the captured image. These adjustments were applied to the entire photograph equally.

**Supplemental Figure 2.**  **Deleting *ef2268* does not impact basal HA digestion.** A whole E. faecalis incubation experiment was performed essentially as described in figure 5A. Lane labels “83.1” and “83.2” are simply different clones of Δ*hylA*Δ*hylB* (pAOJ83), 84 is Δ*hylA*Δ*hylB* (pAOJ84), 55 is Δ*hylA*Δ*hylB* (pAOJ55), Δ3 is the triple mutant in which *hylA*, *hylB* and *ef2268* were deleted sequentially. The blank lane was not inoculated with bacteria. This experiment was performed in duplicate with a representative image shown. Levels were adjusted in Adobe Photoshop for visual clarity and irrelevant portions of the gel were cropped for clarity.

**Supplemental Figure 3. Determination of recombinant HylB concentration and purity**. A dilution series of an unknown concentration of HylB-Myc-6x His and a known concentration of BSA were loaded into a polyacrylamide gel and subjected to electrophoresis. Using the Blue Dry Western method, total protein was stained with Coomassie Brilliant Blue R-250 dye then antibody stained with α-C-Myc 9E10 monoclonal primary and goat anti-mouse HRP conjugated polyclonal secondary antibodies. Two separate monochromatic images were taken with a ChemiDoc MP and combined into a false colored image using the multi-image setting, with chemiluminescence for C-Myc tagged protein being used as the red channel and for total Coomassie Brilliant Blue R-250 stained protein as the blue channel. The raw colorimetric image was used for densitometry with Adobe Photoshop to estimate concentration using a standard curve from the lanes containing BSA. Only lanes of HylB-Myc-6x His that fell within a linear range were used for concentration determination. (A) Monochromatic image taken with the colorimetric setting. (B) Monochromatic image taken with the chemiluminescence setting. (C) Combined false colored image from A and B.

**Supplemental Figure 4. Bladder cytokine and chemokine profiles during *E. faecalis* CAUTI.** Quantification of analytes at 6 (A) and 24 (B) hours post infection in the bladder. Bars show the mean pg/mL values of either two separate experimental replicate pools per bladder (6 hours) or 3 separate experimental pools, per bladder, per infection group (24 hours). ****P*<0.001 by two-way ANOVA with Dunnett’s multiple comparisons between WT and the Δ*hylA*Δ*hylB* strain.
