# Supplementary material for: Function and contribution of two putative Enterococcus faecalis glycosaminoglycan degrading enzymes to bacteremia and catheter-associated urinary tract infection
Source: Infect Immun. 2024 Jun 6;92(7):e00199-24. doi: 10.1128/iai.00199-24 (PMC11238560; doi:10.1128/iai.00199-24)
Supplement: Table S1 — Comparison of bacterial hyaluronidases. [file iai.00199-24-s0006.docx]

| **HylA (EF3023) properties and alignment** | | | | | | | | |
| --- | --- | --- | --- | --- | --- | --- | --- | --- |
| Uniprot ID | Species | Length | Identity | Positive | Gaps | Expect | Mol wt | pI |
| Q837L8 (HylB) | *E. faecalis* | 1004 | 289/792 (36%) | 439/792 (55%) | 51/792 (6%) | 3.0E-152 | 112 kDa | 5.34 |
| P0CZ00 | *P. acnes* | 812 | 170/711 (24%) | 313/711 (44%) | 74/711 (10%) | 8.0E-52 | 88 kDa | 6.73 |
| Q59801 | *S. aureus* | 807 | 212/754 (28%) | 359/754 (47%) | 73/754 (9%) | 6.0E-69 | 92 kDa | 7.24 |
| Q53591 | *S. agalactiae* | 984 | 184/723 (25%) | 347/723 (47%) | 63/723 (8%) | 4.0E-71 | 112 kDa | 8.50 |
| Q54873 | *S. pneumoniae* | 1066 | 219/768 (29%) | 374/768 (48%) | 67/768 (8%) | 4.0E-83 | 121 kDa | 5.81 |
| A0A3D9VCI6 | *T. composti* | 778 | 212/765 (28%) | 361/765 (47%) | 39/765 (5%) | 5.0E-85 | 85 kDa | 5.14 |
| **HylB (EF0818) properties and alignment** | | | | | | | | |
| Uniprot ID | Species | Length | Identity | Positive | Gaps | Expect | Mol wt | pI |
| Q82ZM8 (HylA) | *E. faecalis* | 1372 | 292/793 (37%) | 439/793 (55%) | 53/793 (6%) | 5.0E-155 | 153 kDa | 5.29 |
| P0CZ00 | *P. acnes* | 812 | 182/727 (25%) | 317/727 (43%) | 55/727 (7%) | 9.0E-70 | 88 kDa | 6.73 |
| Q59801 | *S. aureus* | 807 | 218/752 (29%) | 365/752 (48%) | 79/752 (10%) | 9.0E-84 | 92 kDa | 7.24 |
| Q53591 | *S. agalactiae* | 984 | 202/721 (28%) | 354/721 (49%) | 53/721 (7%) | 1.0E-86 | 112 kDa | 8.50 |
| Q54873 | *S. pneumoniae* | 1066 | 215/728 (30%) | 366/728 (50%) | 41/728 (5%) | 2.0E-99 | 121 kDa | 5.81 |
| A0A3D9VCI6 | *T. composti* | 778 | 230/824 (28%) | 382/824 (46%) | 75/824 (9%) | 1.0E-102 | 85 kDa | 5.14 |

**Supplemental Table l.** Protein BLAST alignment and predicted properties^a^ of HylA and HylB vs each other and characterized hyaluronidases from other Gram-positive species.

^a^Both BLAST and property predictions were made using full protein sequence from Uniprot. Properties were predicted with Expasy Protparam.
